# Endoscopic Appearance of Oropharyngeal and Upper GI Kaposi's Sarcoma in an Immunocompromised Patient

**DOI:** 10.1155/2017/3742684

**Published:** 2017-02-05

**Authors:** Umar Darr, Anas Renno, Zubair Khan, Turki Alkully, Maitham A. Moslim, Sehrish Kamal, Ali Nawras

**Affiliations:** University of Toledo Medical Center, Toledo, OH, USA

## Abstract

*Introduction*. Kaposi's sarcoma (KS) usually manifests as a cutaneous disease but GI manifestation is often rare. It is associated with human herpes virus-8 (HHV-8) and seen in immunocompromised patients. In the USA, use of highly active antiretroviral therapy (HAART) has drastically reduced incidence of KS in HIV patients.* Case Presentation*. A 65-year-old male with human immunodeficiency virus (HIV) was admitted to the intensive care unit (ICU) with cardiopulmonary arrest secondary to hyperkalemia of 7.5 meq/L. Following placement of orogastric and endotracheal tube (ETT), a significant amount of blood was noticed in the ETT. Hemoglobin trended down from 9.6 mg/dL to 6.7 mg/dL over five days. Stool guaiac was positive. Esophagogastroduodenoscopy (EGD) was performed and revealed multiple large hypervascularized violaceous submucosal nodular lesions with stigmata of bleeding seen on the soft palate and pharynx and within the cricopharyngeal area close to the vocal cords. Biopsy of the soft palate lesions showed proliferation of neoplastic spindle shaped cells arranged in bundles with slit-like capillary spaces containing erythrocytes consistent with Kaposi's sarcoma. Biopsy was positive for HHV-8. Colonoscopy was unremarkable. There were no cutaneous manifestations of the disease.* Conclusion*. GI involvement of Kaposi's sarcoma must be considered in immunocompromised patients and can be confirmed by endoscopic methods.

## 1. Introduction

Kaposi's sarcoma (KS) usually manifests as slowly progressive multiple reddish purpose cutaneous or mucosal nodules associated with human herpesvirus-8 infection (HHV-8) and seen in immunocompromised patients with some variants [1–3]. Oropharyngeal and upper GI lesions are uncommon.

In the US, the widespread use of highly active antiretroviral therapy (HAART) has profoundly decreased the incidence of KS in HIV-infected patients. On the other hand, the innovation of transplantation and the implication of new generations of immunosuppressants have contributed to the rise of new incidental spikes of KS.

## 2. Case Presentation

**Figure 1:**
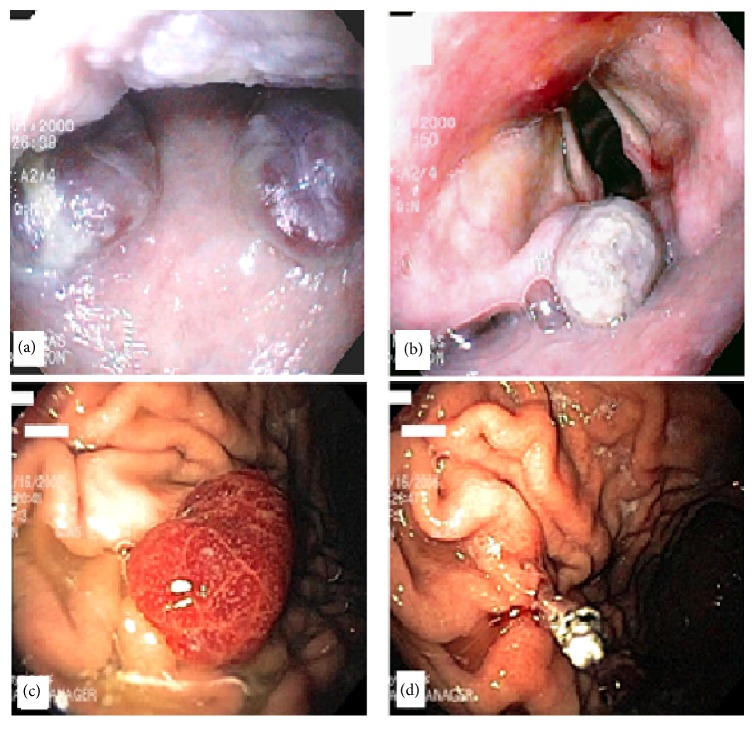
(a) Two hypervascularized violaceous submucosal nodules on soft palate; (b) one submucosal nodule in the oropharynx on the epiglottis near the vocal cords; (c) hypervascularized reddish polyp in the gastric fundus with size of 10 mm; (d) endoclip was placed at postpolypectomy site after removal from polyp seen in image (c).

A 65-year-old Caucasian gentleman with a history of acquired immunodeficiency syndrome (AIDS) on HAART and chronic obstructive pulmonary disease (COPD) was admitted to the intensive care unit (ICU) after having cardiopulmonary arrest secondary to hyperkalemia with a value of 7.5 meq/L (reference range: 3.4–5.2 meq/L). Following the placement of the orogastric tube (OG) and endotracheal tube (ETT), significant amounts of blood were noticed in the ETT. His hemoglobin dropped from 9.6 mg/dL to 6.7 mg/dL over the following five days and his stool was positive for occult blood. He underwent upper GI endoscopy and colonoscopy. Upper endoscopy revealed multiple large hypervascularized violaceous submucosal nodular lesions with stigmata of recent bleeding on the soft palate and pharynx and within the cricopharyngeal area close to the vocal cords. No active bleeding was noted. The examination of the distal esophagus revealed severe ulceration with a small hiatal hernia (see Figures 1(a)–1(d))

A biopsy from the soft palate lesions showed proliferation of neoplastic spindle shaped cells arranged in bundles with slit-like capillary spaces containing erythrocytes consistent with Kaposi's sarcoma. Biopsy was also positive for HHV-8.

Colonoscopy was unremarkable for similar lesions, polyps, or diverticulosis. There were no cutaneous manifestations of the disease. The patient was started on proton pump inhibitors. The bleeding stopped spontaneously without intervention and his hemoglobin stabilized prior to discharge. After his discharge from the hospital, he was scheduled for capsule endoscopy as outpatient to evaluate the small bowel, but unfortunately he expired prior to the exam.

## 3. Discussion

The differential diagnosis of upper GI bleeding for immunocompromised patients is wider than regular patients. Neoplasms are the most frequent cause of upper GI bleeding in patients with AIDS [4]. One of the most common malignancies that can present as GI bleeding is KS, which was widely reported in the 1980s but became less common by the time HAART was used in practice. Recently we saw a new flare of KS due to the administration of new immunosuppression protocols.

Skin lesions are the most common manifestations of KS and are seen in 90% of cases. Other organs in which KS is most frequently involved are lymph nodes, lungs, oropharynx, and the GI tract, particularly the stomach. GI KS may be asymptomatic due to submucosal growth and slow progression of the tumor, or it may express GI bleeding, dysphagia, or location-related pain [5]. During GI endoscopy, KS can manifest as macular angiodysplastic-like lesions [6], vascularized submucosa nodules, mucosal ulcerations, maculopapular lesions, or plaque-like lesions which vary in size [7]. KS may also present in the stomach as linitis plastica. The endoscopic appearance is so typical that, in clinical settings, biopsy may not be necessary [8, 9]. Immunohistologically, HHV-8 detection could be useful to confirm the diagnosis as there is an increased evidence in the literature regarding the association of KS with HHV-8.

## 4. Conclusion

The diagnosis of oropharyngeal and upper GI KS is made via endoscopy. We feel that it is very important for GI endoscopists to be familiar with the endoscopic appearance of KS. In conclusion, immunocompromised patients, including AIDS and transplantation patients, are at risk of developing KS, and at least a few of them might have GI tract involvement. Still routine endoscopic screening for pharyngeal, laryngeal, and GI involvement of KS without apparent symptoms is not recommended, which could be contributed to the fact that the most GI endoscopists are not very familiar with this disease.
